# Intrafamilial Exposure to SARS-CoV-2 Associated with Cellular Immune Response without Seroconversion, France

**DOI:** 10.3201/eid2701.203611

**Published:** 2021-01

**Authors:** Floriane Gallais, Aurélie Velay, Charlotte Nazon, Marie-Josée Wendling, Marialuisa Partisani, Jean Sibilia, Sophie Candon, Samira Fafi-Kremer

**Affiliations:** Strasbourg University Hospital, Strasbourg, France (F. Gallais, A. Velay, C. Nazon, M.-J. Wendling, M. Partisani, J. Sibilia, S. Fafi-Kremer);; Strasbourg University, Strasbourg (F. Gallais, A. Velay, C. Nazon, M.-J. Wendling, J. Sibilia, S. Fafi-Kremer);; Rouen University Hospital, Rouen, France (S. Candon)

**Keywords:** antibodies, coronavirus disease, coronavirus symptoms, COVID-19, household exposure, IgA, IgG, IgM, interferon, intrafamilial contacts, RT-PCR, SARS-CoV-2, serologic testing, severe acute respiratory syndrome coronavirus 2, viral-specific T-cell response

## Abstract

We investigated severe acute respiratory syndrome coronavirus 2 (SARS-CoV-2)–specific antibodies and T-cell responses against SARS-CoV-2 and human coronavirus (HCoV) 229E and OC43 in 11 SARS-CoV-2 serodiscordant couples in Strasbourg, France, in which 1 partner had evidence of mild coronavirus disease (COVID-19) and in 10 unexposed healthy controls. Patients with confirmed COVID-19 were considered index patients and their partners close contacts. All index patients displayed positive SARS-CoV-2–specific antibody and T-cell responses that lasted up to 102 days after symptom onset. All contacts remained seronegative for SARS-CoV-2; however, 6 reported COVID-19 symptoms within a median of 7 days after their partners, and 4 of those showed a positive SARS-CoV-2–specific T-cell response against 3 or 4 SARS-CoV-2 antigens that lasted up to 93 days after symptom onset. The 11 couples and controls displayed positive T-cell responses against HCoV-229E or HCoV-OC43. These data suggest that exposure to SARS-CoV-2 can induce virus-specific T-cell responses without seroconversion.

Coronavirus disease (COVID-19), caused by infection with severe acute respiratory syndrome coronavirus 2 (SARS-CoV-2), is a pandemic that raises a major concern all around the world (*1*). To contain the spread of the virus, several countries have imposed population lockdowns (*2*). In France, the first cases of COVID-19 were recorded at the end of January 2020 (*3*). Due to the rapid increase of new cases and death, a lockdown was imposed during March 17–May 11, 2020. After the lifting of the lockdown, the number of new cases of SARS-CoV-2 decreased substantially. However, we cannot exclude the possibility that a second pandemic wave could occur; an increase in new cases had already been observed in the first week of August 2020 in several regions (*4*). 

Estimating infections with immunizing effects is crucial in helping to predict the postpandemic dynamics of the virus (*4*). Serologic tests for SARS-CoV-2 have been developed to determine the extent of immunity to the virus (*4*), and immunity certifications based on the results of these tests have been considered by some countries in Europe and by the US government. Several persons belonging to households with an index COVID-19 patient reported symptoms of COVID-19 but remained seronegative even though the index patient practiced no quarantine measures. The absence of antiviral antibodies after exposure has been previously reported for other viral infections. In these cases, the presence of virus-specific T-cell responses provided proof of viral transmission (*5*). In this study, we investigated humoral and cellular responses to SARS-CoV-2 in 11 serodiscordant couples in whom 1 partner had evidence of mild COVID-19 and in 10 unexposed healthy blood donors (controls). We also explored the T-cell response against 2 human coronaviruses (HCoV) that cause common colds, given the potential cross-reactive immunity between SARS-CoV-2 and common cold HCoVs. 

## Materials and Methods

### Study Participants

We included in the study 11 couples in whom 1 of the 2 partners met clinical, epidemiologic, and laboratory criteria for a mildly symptomatic confirmed COVID-19 case. We collected blood samples from both partners of each couple during May 7–June 26, 2020. Ten healthy blood donors who had not been exposed to COVID-19 patients and who had tested negative for SARS-CoV-2 antibodies were enrolled as controls. All participants gave written informed consent for research according to protocols approved by the institutional review board of Strasbourg University Hospitals (ClinicalTrials.gov NCT 04405726). 

### SARS-CoV-2 Reverse Transcription PCR

We performed in-house real-time reverse transcription PCR (rRT-PCR) tests for SARS-CoV-2 nucleic acid on samples from nasopharyngeal swab specimens collected during the symptomatic phase from 8 index patients and 3 contacts. Primer and probe sequences target 2 regions of the RdRp gene and are specific to SARS-CoV-2. Assay sensitivity is ≈10 copies/reaction (https://www.who.int/docs/default-source/coronaviruse/real-time-rt-pcr-assays-for-the-detection-of-sars-cov-2-institut-pasteur-paris.pdf). 

### Serologic Tests

We used 3 serologic assays to detect the presence of SARS-CoV-2 antibodies. The Abbott Architect SARS-CoV-2 IgG assay (Abbott, https://www.corelaboratory.abbott) is a chemiluminescent microparticle immunoassay for detecting IgG against the SARS-CoV-2 nucleoprotein and has sensitivity and specificity close to 100% (*6*,*7*). The EUROIMMUN SARS-CoV-2 assay (EUROIMMUN, https://www.euroimmun.com) is an ELISA for detecting IgG and IgA against the SARS-CoV-2 S1 domain of the spike glycoprotein, including the immunologically relevant receptor-binding domain. This assay was reported to have a clinical specificity of 98% for IgG and 91% for IgA detection, with a maximal sensitivity reached after 28 days after symptom onset (IgG 98% and IgA 95%) (*7*). The Biosynex COVID-19 BSS assay (Biosynex, https://www.biosynex.com) is a lateral flow assay for detecting IgM and IgG directed against the SARS-CoV-2 receptor-binding domain of the spike glycoprotein and has a sensitivity of 95.6% and a specificity of 99.4% (*8*). All 3 assays were approved by the French National Agency of Medicine and Health Products Safety for their excellent analytical performances. All tests were performed according to manufacturer instructions. 

### interferon-Gamma Enzyme-Linked Immunospot Assay 

We investigated T-cell immune response against SARS-CoV-2 by performing an interferon-gamma (IFN-γ) enzyme-linked ImmunoSpot ELISPOT assay (ImmunoSpot, http://www.immunospot.com) in duplicate on fresh peripheral blood mononuclear cells (PBMC) isolated from heparin-anticoagulated blood. PBMCs were seeded at 200,000 CD3+ cells/well after dilution according to measurement of CD3+ cell frequencies by flow cytometry. They were stimulated for 20 +4 h with overlapping 15-mer peptide pools used at a final concentration of 1 µg/mL and spanning the sequences of the N-terminal portion of the SARS-CoV-2 spike glycoprotein (pool S1, amino acid residues 1–643) and the C-terminal part of the same protein (pool S2, amino acid residues 633–1273), the nucleoprotein (N), the membrane protein (M), the envelope small membrane protein (E), and the accessory proteins 3A, 7A, 8 and 9B (PepMix; JPT Peptide Technologies, https://www.jpt.com). 

To investigate the possibility of preexisting cross-reactive coronavirus-specific T cells, PBMCs were stimulated in parallel with peptide pools spanning the spike glycoprotein sequences of HCoV-229E (ES1 and ES2) and HCoV-OC43 (OS1 and OS2). Phytohemagglutinin (PHA) was used in duplicate as a positive control and culture medium in quadruplicate as a negative control. After colorimetric revelation of IFN-γ capture (UCytech, https://ucytech.com), spots were counted using an ELISPOT reader (AID, https://www.aid-diagnostika.com). For each condition, the mean number of spot-forming cells per million CD3+ cells was calculated from duplicates after subtraction of the background value obtained from negative controls to determine the frequency of antigen-specific T cells. The threshold defining T-cell reactivity for 1 antigen was set at >3 SD of the negative control background. The SARS-CoV-2-specific T-cell response was considered positive if analysis showed reactivity for >3 SARS-CoV-2 antigens. 

## Results

The median age of the 11 couples was 49 years (range 38–65 years); 11 (50%) were male (Table 1). Partners who met the confirmed case definition of COVID-19 (positive for SARS-CoV-2 by RT-PCR or serology or both) were the first to report symptoms in each couple and were considered index patients (P). Because of the lockdown from March 17 to May 11, 2020, each couple stayed in the same household during this period. Therefore, the partner of each index patient was considered a close contact (C) as defined by the US Centers for Disease Control and Prevention (CDC). 

**Table 1:** Clinical and virological characteristics of COVID-19 patients and their contacts at symptom onset, Strasbourg, France, March 10–26, 2020*

ID	Age, y/sex	RT-PCR†									Duration of symptoms, d	Symptom onset delay, d‡
			Symptoms									
			Fever	Cough	Fatigue	Headache	Anosmia	Agueusia	Dyspnea	Myalgia		
Couples with symptomatic contacts												
P1	47/F	ND	Y	N	Y	Y	Y	Y	N	Y	15	5
C1	50/M	ND	Y	N	N	N	N	N	N	N	3	
P2	54/F	ND	Y	Y	Y	Y	Y		Y	N	13	7
C2	57/M	ND	Y	N	Y	Y	N	N	Y	N	6	
P4§	45/M	Pos (8.39)	Y	Y	N	Y	Y	Y	N	N	12	6
C4	48/F	Neg	Y	Y	N	Y	N	N	N	N	10	
P5	38/M	Pos (7.65)	Y	N	N	Y	Y	Y	N	N	2	10
C5	40/F	Neg	N	Y	N	Y	N	N	N	N	7	
P7	45/M	Pos	Y	Y	N	N	Y	Y	N	Y	4	1
C7	45/F	Neg	N	N	N	N	N	Y	N	N	1	
P8	63/M	Pos	Y	N	Y	Y	Y	Y	N	N	10	10
C8	57/F	ND	N	N	Y	N	N	N	N	Y	10	
Couples with asymptomatic contacts												
P3	65/F	ND	N	Y	N	Y	N	N	N	N	9	NA
C3	61/M	ND	Asymptomatic								NA	
P6#	43/F	Pos (3.99)	N	Y	N	N	Y	Y	Y	N	7	NA
C6	45/M	ND	Asymptomatic								NA	
P9	57/M	Pos (6.20)	Y	Y	N	N	N	N	Y	Y	14	NA
C9	58/F	ND	Asymptomatic								NA	
P10	39/F	Neg	N	N	N	Y	Y	Y	N	N	10	NA
C10	39/M	ND	Asymptomatic								NA	
P11	58/F	Pos (8.46)	Y	N	Y	Y	N	N	N	N	21	NA
C11	57/M	ND	Asymptomatic								NA	

*In ID column, same number indicates partners in 1 couple. C, contact; d, days; ID, identification; ND, not done; NA, not applicable; neg, negative; P, index patient; pos, positive; RT-PCR, reverse-transcription PCR; SARS-CoV-2, severe acute respiratory syndrome coronavirus 2. †SARS-CoV-2 RT-PCR was performed in nasopharyngeal specimens during the symptomatic phase. When available, viral load (log copies/reaction) is indicated in parentheses below the result. ‡Days from symptom onset in index patient to onset in contact. §This index patient quarantined himself by dining separately and wearing a mask after positive SARS-CoV-2 PCR testing 1 day after symptom onset. #This index patient wore a mask after positive SARS-CoV-2 PCR testing 3 days after symptom onset.

During March 2–April 9, all index patients reported histories of >1 symptoms: 8 had fever, 6 had cough, 4 had fatigue, 8 had headache, 8 had anosmia, 7 had ageusia, 3 had dyspnea, and 3 had myalgia (Table 1). We tested 8 of these patients for SARS-CoV-2 using RT-PCR on nasopharyngeal samples; results for 7 were positive (Table 1). The duration of symptoms varied (2–21 days, median 10 days). During this symptomatic phase, all couples rigorously washed their hands, and each avoided hugs and kisses with his or her partner except couple 2. Nine of the 11 couples slept in the same bed. Only 2 index patients, P4 and P6 (i.e., the index partners from couples 4 and 6), quarantined themselves by eating and sleeping separately or wearing a mask or both for 1 day (P4) and 3 days (P6) after symptom onset. 

We performed serologic testing for SARS-CoV-2 antibodies in index patients at a median of 68 days (range 49–102 days) after symptom onset. All displayed IgG against the SARS-CoV-2 N protein, the spike glycoprotein, or both, as indicated by the 3 serologic assays (Table 2), confirming the persistence of the SARS-CoV-2 antibodies for up to 102 days after symptom onset. Results of tests for SARS-CoV-2 IgA were positive for 7 of the 11 index patients (Table 2). 

**Table 2:** Humoral and cellular immune response to SARS-CoV-2 of COVID-19 patients and their contacts 44–102 days after symptom onset, Strasbourg, France, 2020*

ID†	Lymphocyte count, × 10^9^/L	Days from symptom onset to sample collection	SARS-CoV-2 serologic test result	Assay results						SARS-CoV-2–specific T-cell response (no. antigens)
				Biosynex Antigen: RBD of protein S			Abbott Architect Antigen: protein N (index value)		Euroimmun Antigen: protein S (index value)	
IgM	IgG	IgG	IgG/IgA
Couples with symptomatic contacts										
P1	1.3	58	Pos	Neg	Pos		Pos (3.36)		Pos (2.28)/neg	Pos (5)†
C1	1.8	53	Neg	Neg	Neg		Neg		Neg/neg	Pos (4)†
P2	2.0	51	Pos	Neg	Pos		Pos (4.3)		Pos (2.3)/neg	Pos (7)†
C2	1.5	44	Neg	Neg	Neg		Neg		Neg/neg	Neg (1)
P4	1.6	57	Pos	Pos	Pos		Pos (6.48)		Pos (4.24)/pos (4.16)	Pos (4)†
C4	1.7	51	Neg	Neg	Neg		Neg		Neg/neg	Pos (3)†
P5	1.6	68	Pos	Pos	Pos		Pos (3.97)		Pos (4.86)/pos (2.38)	Pos (7)†
C5	2.0	80	Neg	Neg	Neg		Neg		Neg/neg	Pos (3)†
P7	1.3	64	Pos	Pos	Neg		Pos (4.18)		Pos (3.43)/doubtful (0.94)	Pos (4)†
C7	2.2	64	Neg	Neg	Neg		Neg		Neg/neg	Neg (1)
P8	1.9	102	Pos	Pos	Pos		Pos (7.55)		Pos (5.39)/pos (9.23)	Pos (5)†
C8	1.9	93	Neg	Neg	Neg		Neg		Neg/neg	Pos (4)†
Couples with asymptomatic contacts										
P3	1.7	49	Pos	Pos	Pos		Pos (8.4)		Pos (7.23)/pos (3.83)	Pos (8)†
C3	1.5	NA	Neg	Neg	Neg		Neg		Neg/Neg	Neg (0)
P6	2.1	69	Pos	Pos	Pos		Pos (6.37)		Pos (5.73)/pos (2.49)	Pos (6)†
C6	2.2	NA	Neg	Neg	Neg		Neg		Neg/doubtful (0.85)	Neg (1)
P9	2.4	88	Pos	Pos	Pos		Pos (7.48)		Pos (7.02)/pos (5.98)	Pos (6)†
C9	3.0	NA	Neg	Neg	Neg		Neg		Neg/neg	Neg (1)
P10	1.9	99	Pos	Pos	Neg		Pos (2.46)		Pos (1.75)/doubtful (0.95)	Pos (5)†
C10	1.1	NA	Neg	Neg	Neg		Neg		Neg/neg	Neg (1)
P11	1.7	100	Pos	Neg	Pos		Neg		Pos (4.01)/pos (1.72)	Pos (6)†
C11	1.9	NA	Neg	Neg	Neg		Neg		Neg/neg	Neg (0)

*In ID column, same number indicates P and C are partners in 1 couple. C, contact; ID, identification; neg, negative; P, index patient; pos, positive; PBMC, peripheral blood mononuclear cells; NA, not applicable; RBD, receptor-binding domain; SARS-CoV-2, severe acute respiratory syndrome coronavirus 2.

Six of the 11 contacts (C1, C2, C4, C5, C7, and C8) experienced symptoms 1–10 days after symptom onset in their partners (Table 1). We tested 3 of them for SARS-CoV-2 RNA by RT-PCR on samples from nasopharyngeal swab specimens during the symptomatic phase; results for all were negative (Table 1). Three had fever, 2 had cough, 2 had fatigue, 3 had headache, 1 had ageusia, 1 had dyspnea, and 1 had myalgia. The duration of symptoms varied (1–10 days, median 7 days) (Table 1). We performed serologic testing for SARS-CoV-2 at a median of 59 days (range 44–93 days) after symptom onset in symptomatic contacts and at the same time as their partners for asymptomatic contacts. All the contacts, including the symptomatic ones, were SARS-CoV-2 seronegative for IgM, IgA (except 1 equivocal result), and IgG (Table 2). 

**Figure 1:**
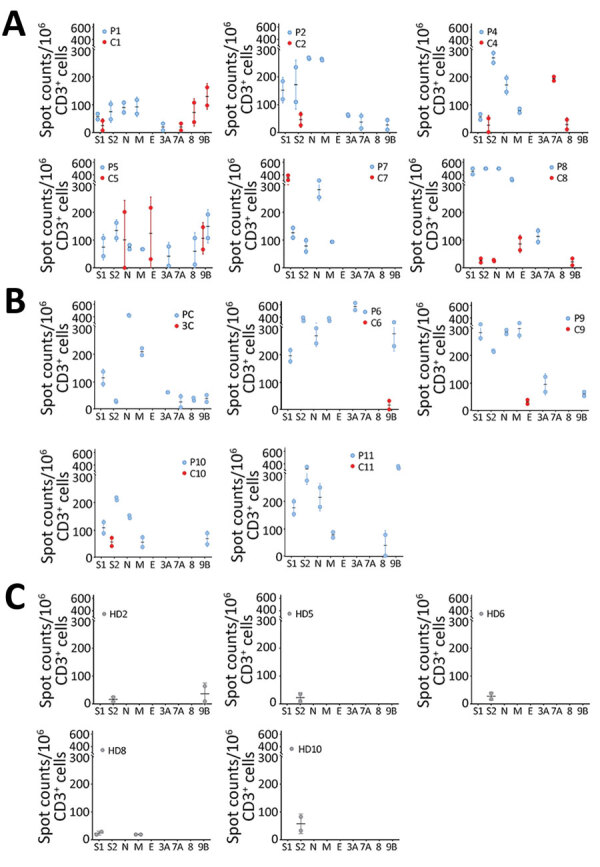
SARS-CoV-2–specific T-cell response patterns in index patients, contacts, and unexposed healthy donors in study of intrafamilial exposure to SARS-CoV-2, France. A, B) Spot counts of SARS-CoV-2–specific T cells measured by interferon-gamma (IFN-γ) ELISPOT assay are shown for 11 couples, each including 1 confirmed coronavirus disease case (P) and 1 SARS-CoV-2 seronegative symptomatic (A) or asymptomatic (B) contact (C). C) Spot counts of IFN-γ–producing T cells in response to SARS-CoV-2 antigens are shown for the 5 out of the 10 controls (HD) tested who displayed detectable T-cell responses. All experiments were performed in duplicate. Data are shown as means and standard deviations of spots counts of IFN-γ–producing T cells per 1 million CD3+ cells. Each dot represents a single measured value. Blue dots correspond to T-cell responses detected in index patients, red dots correspond to those detected in contacts and gray dots to those found in healthy donors. The x-axis represents the SARS-CoV-2 antigens spanned by the peptide pools used in ELISPOT assays: the N-terminal and C-terminal regions of SARS-CoV-2 spike glycoprotein (S1 and S2, respectively); the N, M, and E proteins; and the accessory proteins 3A, 7A, 8, and 9B. C, contact; E, envelope small membrane protein; HD, healthy blood donor (control); M, membrane protein; N, nucleoprotein; P, index patient; SARS-CoV-2, severe acute respiratory syndrome coronavirus 2.

**Figure 2:**
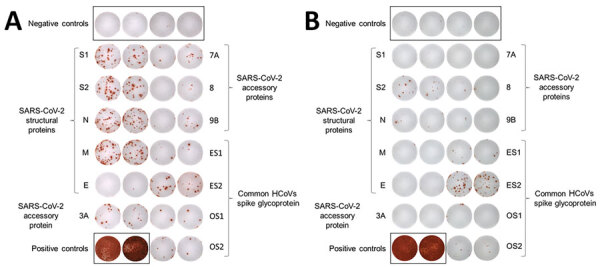
Example of IFN-γ ELISPOT images corresponding to couple 2 (P2 and C2) in a study of intrafamilial exposure to SARS-CoV-2, France. T-cell–specific response was evaluated using peptide pools spanning SARS-CoV-2 structural protein (spike glycoprotein: N-terminal region = S1, C-terminal region = S2; N, M, and E proteins); SARS-CoV-2 accessory proteins (3A, 7A, 8, and 9B); and the N-terminal and C-terminal regions of the spike glycoprotein of common cold human coronaviruses 229 (ES1 and ES2) and OC43 (OS1 and OS2). All experiments were performed in duplicate with 4 wells of negative controls (cells with culture medium only) and 2 wells of positive controls (phytohemagglutinin) for each individual. P2 was reactive to all antigens tested except for SARS-CoV-2 proteins E and 8, whereas C2 was reactive to 1 SARS-CoV-2 protein only (S2) and to ES1, ES2, and OS2. C, contact; E, envelope small membrane protein; HCoV, human coronavirus; IFN-γ, interferon gamma; M, membrane protein; N, nucleoprotein; P, index patient; SARS-CoV-2, severe acute respiratory syndrome coronavirus 2.

To investigate the SARS-CoV-2–specific T-cell response in the 11 couples, we collected fresh PBMC samples on the same day as the serum collections. We then stimulated the samples with 4 structural and 4 accessory SARS-CoV-2 proteins followed by IFN-γ ELISPOT analysis. All index and contact patients had normal lymphocyte counts (Table 2). All index patients showed SARS-CoV-2–specific IFN-γ responses against 4–8 SARS-CoV-2 antigens (Table 2; Figure 1). All of their immune systems recognized the structural proteins S1, S2, N, and M, and 9 of them recognized >1 accessory protein (3A, 7A, 8, or 9B), showing that SARS-CoV-2–specific T-cell responses had developed (Figures 1,2; Appendix Figure 1). Blood samples were collected 49–102 days after symptom onset, which suggests that antiviral T cells are maintained for up to 102 days in patients having recovered from mild COVID-19. 

We evaluated SARS-CoV-2–specific T-cell response in contacts at a median time of 59 days (range 44–93 days) after symptom onset in symptomatic contacts and at the same time as their partner for asymptomatic contacts. Among the 6 symptomatic contacts, 4 (C1, C4, C5, and C8) displayed a positive SARS-CoV-2–specific T-cell response with a reactivity to >3 SARS-CoV-2 antigens (Figure 1, row A; Appendix Figure 1). Contact C1 exhibited T-cell reactivity against 4 SARS-CoV-2 antigens, including 1 structural protein (S1) and 3 accessory proteins; contact C5 exhibited T-cell reactivity against 2 and C8 against 3 structural proteins (N, E, and S2 for C8) and the accessory protein 9B. Contact C4 exhibited T-cell reactivity against 1 structural protein (S2) and 2 accessory proteins. Although symptomatic contact C7 exhibited T-cell SARS-CoV-2–specific response against a single antigen (structural protein S1), the frequency of IFN-γ–producing T cells was higher than that observed in his partner (mean 353 + 53 vs. 126 + 25 spot-forming units/1 million cells). Symptomatic contact C2 and asymptomatic contacts C6, C9, and C10 exhibited a low frequency of T-cell reactivity against a single antigen (S2 = 2, E = 1, 9B = 1) that was not considered here as a positive specific T-cell response to SARS-CoV-2 (Figure 1, row A and B; Figure 2; Appendix Figure 1). The asymptomatic contacts C3 and C11 showed no T-cell response against any of the SARS-CoV-2 antigens (Figure 1, row B; Appendix Figure 1). 

We included 10 unexposed HD as controls, with a mean age of 46 years (range 29–60 years). We confirmed their SARS-CoV-2 seronegative status with the 3 serologic assays. Five of them displayed low T-cell reactivity to SARS-CoV-2 against 1 or 2 antigens (S1, S2, M, 9B) (Figure 1, row C; Appendix Figure 1). 

**Figure 3:**
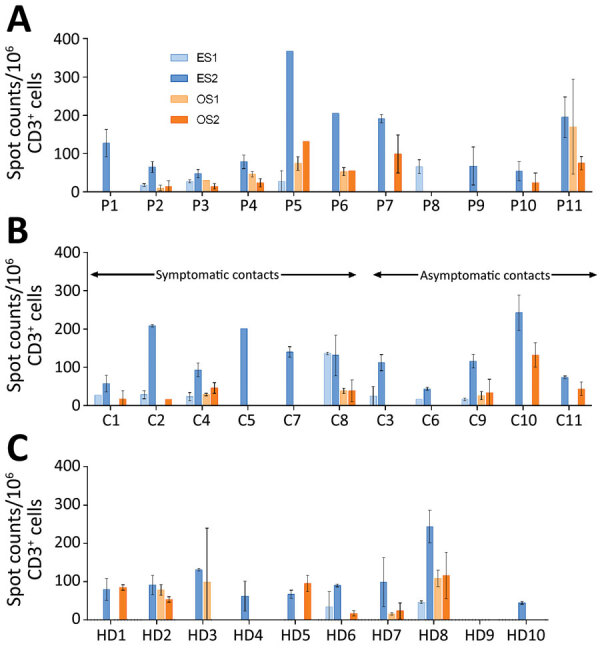
Frequency of specific T cells directed against spike glycoprotein antigens of the 2 common cold HCoVs 229E and OC43 in study of intrafamilial exposure to SARS-CoV-2, France. A) Index patients (n = 11); B) seronegative partners of index patients (n = 11); C) unexposed healthy controls (n = 10). Spot counts of common cold human coronaviruses-specific T cells were measured by interferon-gamma ELISPOT assay. All experiments were performed in duplicate. Data are shown as means and standard deviations of spot counts of interferon-gamma–producing T cells per 1 million CD3+ cells. T-cell secretion of IFN-γ was determined in response to peptide pools spanning the N-terminal and the C-terminal regions of the spike glycoprotein of HCoV 229E (ES1 and ES2 subpools) and HCoV OC43 (OS1 and OS2 subpools). Each color corresponds to 1 antigen subpool. C, contact; HCoV, human coronavirus; HD, healthy blood donor (control); P, index patient; SARS-CoV-2, severe acute respiratory syndrome coronavirus 2.

**Figure 4:**
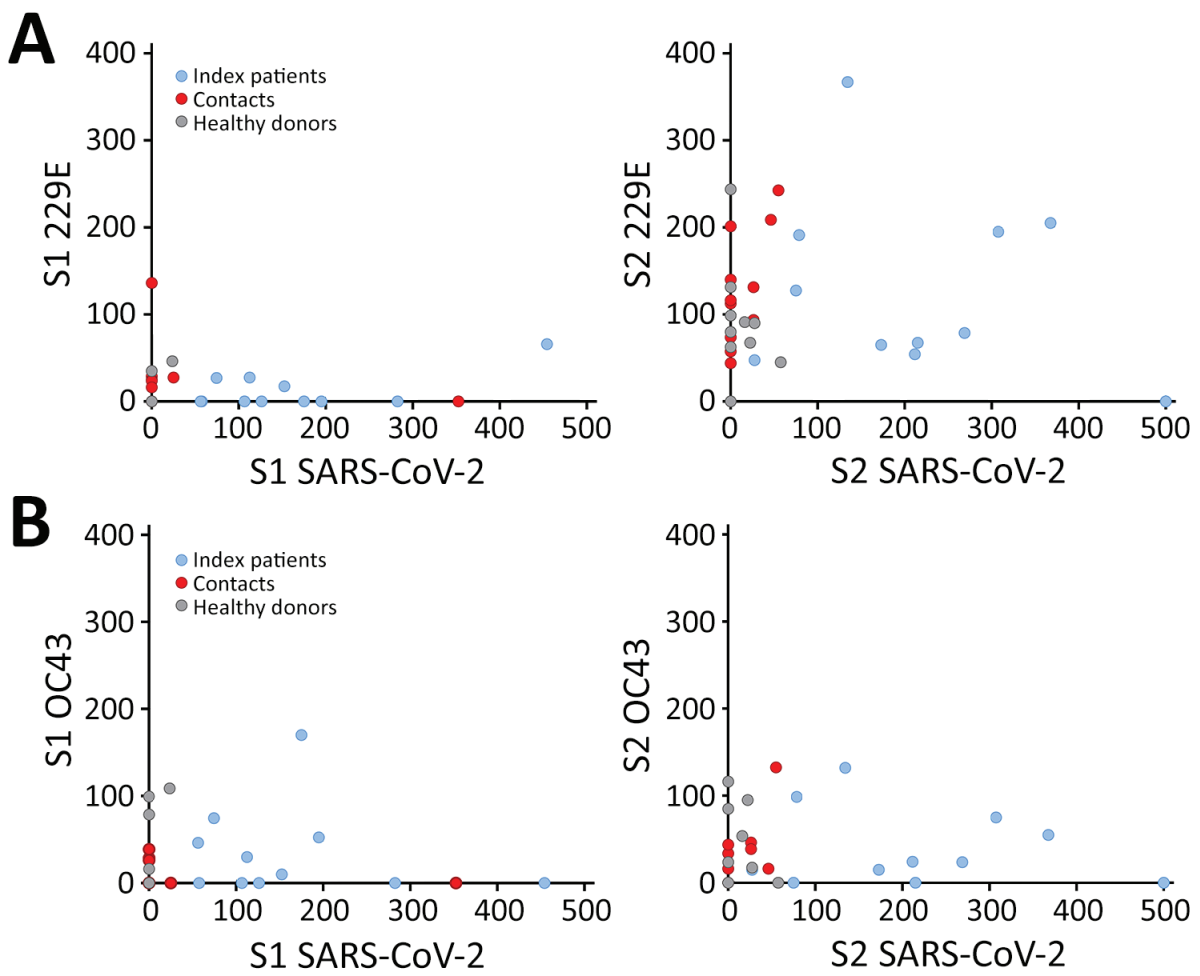
Correlation of the T-cell responses against spike glycoprotein antigens of SARS-CoV-2 and HCoVs 229E and OC43 in study of intrafamilial exposure to SARS-CoV-2, France. Means of spot counts of interferon gamma–producing T cells per 1 million CD3+ cells in response to peptide pools spanning the N terminal (S1) and the C-terminal (S2) regions of spike glycoproteins of SARS-CoV-2 compared with HCoV-229E (A) and HCoV-OC43 (B) in 11 confirmed coronavirus disease cases (index patients), their seronegative partners (contacts), and 10 healthy blood donor controls. HCoV, human coronavirus; SARS-CoV-2, severe acute respiratory syndrome coronavirus 2.

A recent study demonstrated that several CD4 T cells reacting to SARS-CoV-2 epitopes were a result of a cross-reaction with corresponding homologous sequences from commonly circulating HCoVs including OC43 and 229E, which can cause common colds (*9*). To investigate if there was a correlation between T-cell responses against SARS-CoV-2 and common cold HCoVs, we tested the 11 couples and the 10 unexposed controls for reactivity against the spike glycoprotein (S1 and S2 regions) of HCoV-229E and HCoV-OC43. All but 1 HD (HD9) showed IFN-γ–producing T cells directed against these antigens (Figure 3; Appendix Figure 2). Eight index patients (P2, P3, P4, P5, P6, P7, P10, and P11), 7 contacts (C1, C2, C4, C8, C9, C10, and C11), and 7 controls displayed a positive T-cell response against both HCoV-229E and HCoV-OC43. Three index patients (P1, P8, and P9), 4 contacts (C3, C5, C6, and C7), and 2 controls displayed positive T-cell responses only against HCoV-229E. We found no correlation between the responses to S1 and S2 peptide pools of SARS-CoV-2 and HCoVs (Figure 4). 

## Discussion 

In this study, we demonstrate that intrafamilial contacts can display a SARS-CoV-2–specific T-cell response in the absence of seroconversion, especially when they have been symptomatic. This T-cell response provides evidence that transient or anatomically contained SARS-CoV-2 infection, or both, may have occurred and that T-cell responses would be more sensitive indicators of SARS-Co-V-2 exposure than antibodies.

Each couple stayed in the same household during the COVID-19 episode and the partners were in close contact for a long time due to the lockdown. Although 5 contacts were asymptomatic, 6 exhibited symptoms a median of 7 days after symptom onset in their partners, suggesting that at least those 6 were infected. However, results from neither RT-PCR nor serology testing using 3 different assays and targeting 2 different SARS-CoV-2 structural proteins were positive in contacts. In contrast, analysis of SARS-CoV-2–specific T-cell response showed a positive response against >3 antigens, including structural proteins in 4 symptomatic contacts, strongly suggesting that they were infected with SARS-CoV-2. 

Five unexposed controls and 1 symptomatic and 3 asymptomatic contacts exhibited low frequencies of SARS-CoV-2 IFN-γ–producing T cells. Because these 4 contacts were exposed to COVID-19 patients and the unexposed controls donated blood in April and May 2020, it is unclear whether the detectable T-cell responses were the result of cross-reactivity with common cold HCoV antigens, as previously reported (*10**–**12*) or of SARS-CoV-2 infection. Although recent research provided direct evidence of cross-reactivity between SARS-CoV-2 epitopes and common cold HCoVs (*9*), we observed no obvious relationship between the magnitude of T-cell responses against spike glycoproteins of common cold HCoVs and SARS-CoV-2 in index patients, contacts, and unexposed HD. In parallel with our findings, another recent study (*13*) reported finding memory T-cell response against SARS-CoV-2 structural proteins in exposed family members and healthy persons lacking detectable circulating antibodies who donated blood during the pandemic. 

There are multiple explanations for virus-specific T cells developing without any antibody response. A study in a small cohort of patients (*14*) reported that 40% of asymptomatic and 12.9% of patients with mild COVID-19 no longer had antibodies 56 days after being discharged from the hospital. In our study, the serum samples were collected between 49 to 102 days after symptom onset, so it is possible that the contacts had lost their antibodies during this period. It is also possible that very low levels of antibodies that might have developed in contacts were not detected by the serologic assays we used. The lack of specific antibodies might also be because of exposure to low doses of the virus with brief and transient viral replication, to a downstream event of protective innate immune response, or to abortive replication of defective viral genomes (*5*).

Eventually, the presence of SARS-CoV-2-specific T-cell response, whether because of infection with SARS-CoV-2 or a cross-reaction, might explain the mild and rapidly resolved symptoms in index patients and symptomatic contacts and the resistance of other contacts to symptomatic SARS-CoV-2 infection. However, this possible explanation needs to be investigated further in a large cohort. 

Our study is subject to several limitations. First, our findings suffer from a limited sample size, although this is a unique cohort, and it was not possible to increase the sample size. Second, because of the unavailability of PBMCs collected before the pandemic, we recruited unexposed HD who donated their blood during the pandemic as controls, so we cannot exclude a potential infection by SARS-CoV-2 before the enrollment in the study. Third, although we detected high frequencies of T-cell response against diverse SARS-CoV-2 proteins in symptomatic contacts lacking circulating antibodies, it remains possible that a part of this response may be a result of cross-reaction with common cold HCoVs. 

Overall, our results indicate that persons exposed to SARS-CoV-2 may develop virus-specific T-cell responses without detectable circulating antibodies. This aspect of the immune response against SARS-CoV-2 contributes substantially to the understanding of the natural history of COVID-19. Furthermore, our data indicate that epidemiologic data relying solely on the detection of SARS-CoV-2 antibodies may lead to a substantial underestimation of prior exposure to the virus. Our data may also have implications for vaccine development and tracking the future evolution of the SARS-CoV-2 pandemic. 

Appendix Additional information on intrafamilial exposure to SARS-CoV-2 associated with cellular immune response without seroconversion, France.
