# Supplementary material for: Intrafamilial Exposure to SARS-CoV-2 Associated with Cellular Immune Response without Seroconversion, France
Source: Emerg Infect Dis. 2021 Jan;27(1):113–21. doi: 10.3201/eid2701.203611 (PMC7774579; doi:10.3201/eid2701.203611)
Supplement: Appendix — Additional information on intrafamilial exposure to SARS-CoV-2 associated with cellular immune response without seroconversion, France. [file 20-3611-Techapp-s1.pdf]

Article DOI: https://doi.org/10.3201/eid2701.203611

Intrafamilial Exposure to SARS-CoV-2 Associated with Cellular Immune Response without Seroconversion, France

Appendix

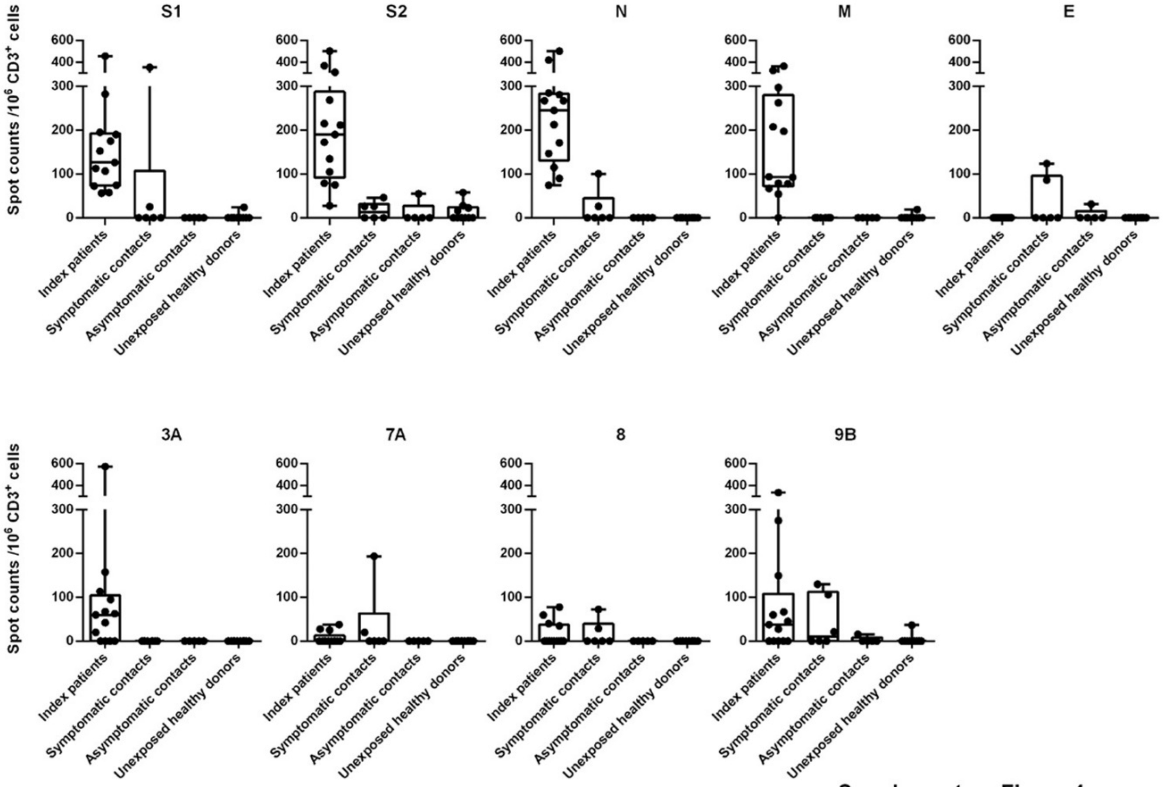

Appendix Figure 1. Specific T-cell response against structural and accessory SARS-CoV-2 proteins evaluated by ELISPOT assays. Dot plots showing the frequencies of IFNγ-producing cells responding in ELISPOT assays to overlapping peptides spanning several SARS-CoV-2 structural proteins [N- and C-terminal parts of spike protein (S1 and S2, respectively), nucleocapside (N), membrane (M), envelope (E)] and accessory proteins (7A, 8, and 9B). Each dot represents the mean response of 1 individual. Four groups are represented: COVID-19 confirmed cases (index patients), their partners with or without symptoms developed in the days after onset in the index patient, and unexposed healthy donors.

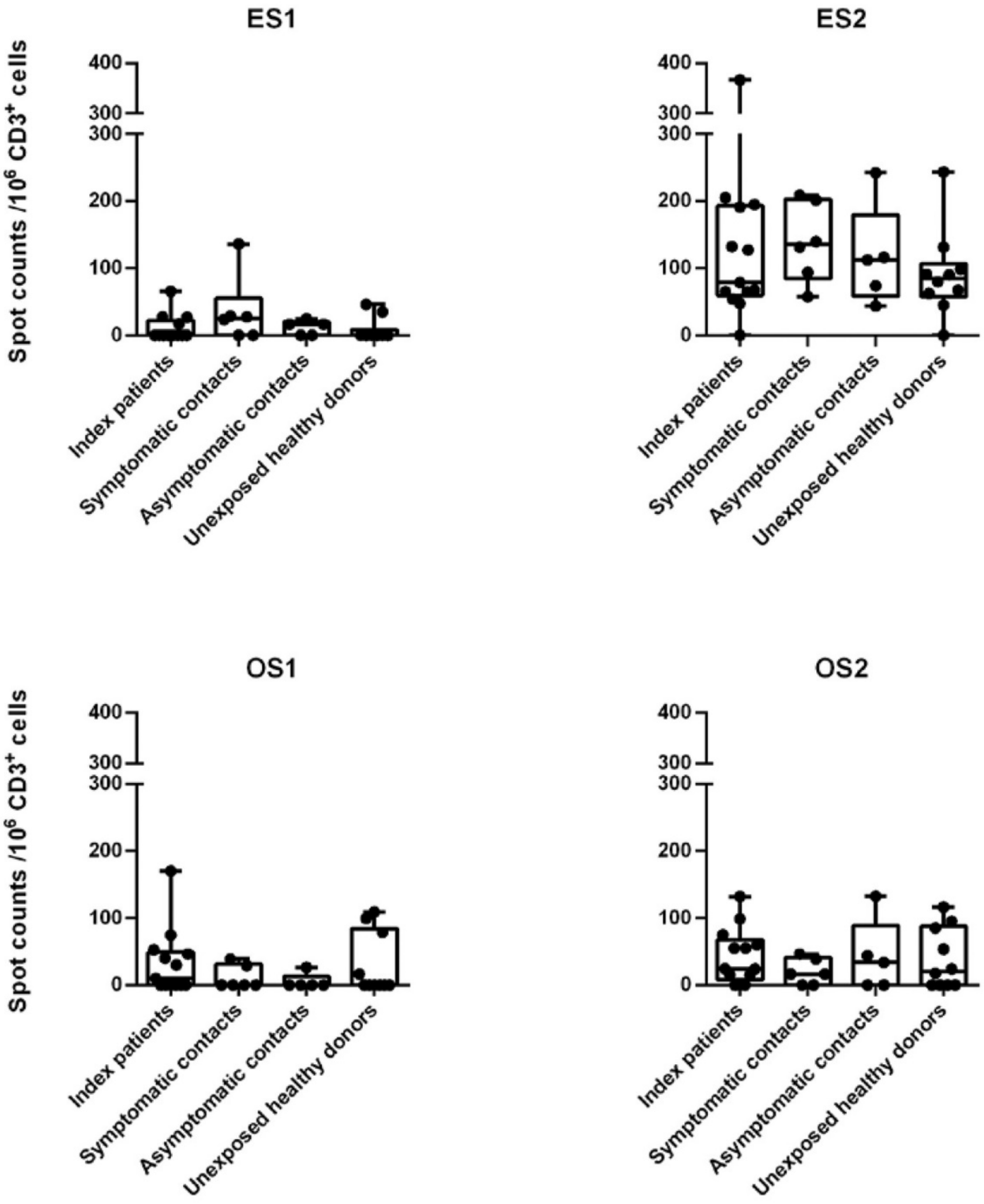

Appendix Figure 2. Specific T cells against the spike protein of the human coronaviruses HCoV-229E and OC43 evaluated by ELISPOT assays. Dot plots showing the frequencies of IFNγ-producing cells responding in ELISPOT assays to overlapping peptides spanning the N- and the C-terminal parts of the spike protein of the human coronaviruses HCoV-229E (ES1 and ES2, respectively) and OC43 (OS1 and OS2, respectively). Each dot represents the response of 1 individual. Four groups are represented: COVID-19 confirmed cases (index patients), their partners with or without symptoms developed in the days after onset in the index patient, and unexposed healthy donors.
